# Persisting Motor Function Problems in School-Aged Survivors of Congenital Diaphragmatic Hernia

**DOI:** 10.3389/fped.2021.729054

**Published:** 2021-10-27

**Authors:** Sophie de Munck, Monique H. M. van der Cammen-van Zijp, Tabitha P. L. Zanen-van den Adel, René M. H. Wijnen, Suzan C. M. Cochius-den Otter, Neeltje E. M. van Haren, Saskia J. Gischler, Joost van Rosmalen, Hanneke IJsselstijn

**Affiliations:** ^1^Department of Pediatric Surgery and Pediatric Intensive Care, Erasmus MC Sophia Children's Hospital, Rotterdam, Netherlands; ^2^Department of Orthopedics, Section of Physical Therapy, Erasmus MC Sophia Children's Hospital, Rotterdam, Netherlands; ^3^Department of Child and Adolescent Psychiatry and Psychology, Erasmus MC Sophia Children's Hospital, Rotterdam, Netherlands; ^4^Department of Biostatistics, Erasmus MC, Rotterdam, Netherlands; ^5^Department of Epidemiology, Erasmus MC, Rotterdam, Netherlands

**Keywords:** congenital diaphragmatic hernia, motor function, development, critical illness, follow-up

## Abstract

**Background and Objectives:** Children born with congenital diaphragmatic hernia (CDH) and treated with extracorporeal membrane oxygenation (ECMO), are at risk for motor function impairment during childhood. We hypothesized that all children born with CDH are at risk for persistent motor function impairment, irrespective of ECMO-treatment. We longitudinally assessed these children's motor function.

**Methods:** Children with CDH with and without ECMO-treatment, born 1999–2007, who joined our structural prospective follow-up program were assessed with the Movement Assessment Battery for Children (M-ABC) at 5, 8, 12 years. Z-scores were used in a general linear model for longitudinal analysis.

**Results:** We included 55 children, of whom 25 had been treated with ECMO. Forty-three (78%) were evaluated at three ages. Estimated mean (95% CI) z-scores from the general linear model were −0.67 (−0.96 to −0.39) at 5 years of age, −0.35 (−0.65 to −0.05) at 8 years, and −0.46 (−0.76 to −0.17) at 12 years. The 5- and 8-years scores differed significantly (*p* = 0.02). Motor development was significantly below the norm in non-ECMO treated patients at five years; −0.44 (−0.83 to −0.05), and at all ages in the ECMO-treated-patients: −0.90 (−1.32 to −0.49), −0.45 (−0.90 to −0.02) and −0.75 (−1.2 to −0.34) at 5, 8, and 12 years, respectively. Length of hospital stay was negatively associated with estimated total z-score M-ABC (*p* = 0.004 multivariate analysis).

**Conclusion:** School-age children born with CDH are at risk for motor function impairment, which persists in those who received ECMO-treatment. Especially for them long-term follow up is recommended.

## Introduction

Congenital diaphragmatic hernia (CDH) is a rare anomaly with a prevalence of 2.3 per 10,000 births (1). This anomaly brings along a broad spectrum of problems, of which lung hypoplasia and pulmonary hypertension contribute considerably to the mortality rate of ~28% (2). Over the last decade, improved treatment of CDH has increased the survival rates as well as the prevalence of long-term morbidity (1, 3). The latter may last into adolescence and beyond, affecting several domains of development (4, 5). Among all domains, motor impairment is frequently reported, and gross motor function deficits in particular (3). This is of concern, given that motor impairments may affect the child's life on multiple levels, such as not being fully able to participate in sports and lagging behind one's peers. The underlying cause of these impairments remains unknown; yet, a number of determinants have been suggested, disease- as well as treatment-related (6–8). Nevertheless, the identification of specific risk factors might help to develop a tool for risk stratification. Survivors of CDH are already at risk for motor delay in the first years of life (9), although contradictory results on motor development in toddlers born with CDH have been published (10, 11). Aged 5 and 8 years, children born with CDH have been found to be at risk for impaired motor function, regardless of having received extracorporeal membrane oxygenation (ECMO) treatment (6, 12). In a longitudinal study concerning motor development in children with a variety of diagnoses treated with ECMO, the ones diagnosed with CDH appeared to be at the highest risk of impaired motor function (8). However, the course of motor function impairment over time in the complete spectrum of CDH-survivors is yet to be discovered (3). We hypothesized that children born with CDH are at risk for longitudinal motor function impairment. We longitudinally studied motor function, establishing total scores on motor function as well as subskills scores, together with its determinants, in a population of CDH-survivors treated either with or without ECMO.

## Methods

### Patients

All children born with CDH between January 1999 and November 2007, and who had joined our prospective follow-up program in the Erasmus MC Sophia Children's Hospital, were included. This program involves assessment by an experienced pediatric physical therapist at ages 30 months, 5, 8, 12, and 17 years, including motor performance up till 12 years of age, and exercise capacity up till 17 years of age (6, 8, 13). In case of emerging motor problems, children are offered extra help; e.g., referral for physical therapy. For this study, we analyzed motor function outcomes at 5, 8, and 12 years of age. The children included in the study had all undergone at least one assessment of motor function. For organizational reasons, the assessment at 12 years of age was discontinued for non-ECMO treated patients between 2011 and 2013. Data were collected until January 2020.

### Exclusion Criteria

Children were excluded if they had been diagnosed with CDH later than seven days post-partum or when the anomaly appeared to be a para-esophageal hernia or a diaphragmatic eventration. Children who could not be reliably assessed, e.g. those with a chromosomal disorder known to affect motor performance or with severe neurodevelopmental impairment, were excluded as well. As ruled by the Erasmus MC Medical Ethics Review Board, this study was exempt from the Dutch Medical Research Involving Human Subjects Act. Therefore, Medical Ethics Review Board approval was waived (MEC-2020-0551). Data acquisition took place as part of routine clinical care. Parents of included children were informed that data were evaluated.

### Motor Function Assessment

Both the first and the second version of the Movement Assessment Battery for Children (M-ABC), validated for children from 3 to 16 of age, were used to assess motor performance (14, 15). The original norm scores and cutoff values are applicable to Dutch children (15). Between March 2004 and October 2012, we used the first version of the M-ABC, and from November 2012 onwards the M-ABC-2. From here on, the term M-ABC refers to both versions, whose content is similar and assumed to be comparable (16, 17). The M-ABC is divided in three age bands. Each band contains age-appropriate tests covering three domains: manual dexterity (3 items), ball skills (2 items) and balance skills (3 items).

### Characteristics

We recorded the following perinatal characteristics: gender, birthweight (grams), gestational age (weeks), inborn (yes/no; yes if born in our hospital or other CDH center), prenatal diagnosis (yes/no), side of the defect, age at surgery (days), primary closure of defect (yes/no), duration of initial ventilation (days), duration of initial stay at the pediatric intensive care unit (PICU) (days), duration of initial hospital stay (days), cardiac malformations (yes/no; recorded if follow up by a pediatric cardiologist was necessary), treatment with inhaled nitric oxide (yes/no), chronic lung disease (CLD) (18), sepsis during initial hospital stay (defined as clinical suspicion confirmed with positive blood culture), ECMO-treatment (yes/no), age at start ECMO (hours), and duration of ECMO-treatment (hours), and maternal education level as classified by the International Standard Classification of Education (19).

At follow-up, the following characteristics were collected: weight-for-height z-score (20), having obtained a swimming certificate, sports participation (yes/no, yes if at least once a week, other than gymnastics at school). All characteristics were retrieved from electronic patient files.

### Statistical Analysis

Mann-Whitney U tests were used to compare continuous data of participants and non-participants, who were lost to follow up, as well as data of ECMO-treated and non-ECMO treated patients; chi-square tests were used for categorical data. All statistical tests were two-sided with a significance level of 0.05.

Raw scores from the M-ABC assessment were converted to percentile scores for clinical interpretation. For both versions of the M-ABC, a score equal to or below the fifth percentile indicates a definite motor problem. Therefore, we classified children as those who have a definite motor problem and those who have not (14, 15). The chi-square test was used to compare outcome proportions in our sample of CDH patients with normative proportions. To combine scores of both M-ABC versions, the analysis was based on the percentile scores of the M-ABC, and a probit transformation (i.e., inverse normal transformation) was performed to transform the percentile scores into z-scores (16).

For the longitudinal analysis of motor development over the years, we used general linear models (GLM) for repeated measurements. An advantage of GLM is that it accounts for data that are missing at random. The dependent variable in this model is the z-scores of the M-ABC. Age (coded as a categorical variable), ECMO treatment (yes/no) and their interaction effect were included as independent variables. Mean values of the M-ABC were compared between age groups, for the entire sample and stratified by ECMO group, using the estimated marginal means of this model. The determinants birthweight, CLD, initial hospital stay, primary closure of defect, sports participation and weight-for-height z-score at follow up were added to this model as independent variables, first one by one for univariate analyses and eventually all together for multivariate analysis. An unstructured covariance matrix was assumed to account for the within-patient correlations between the three age groups.

## Results

**Figure 1:**
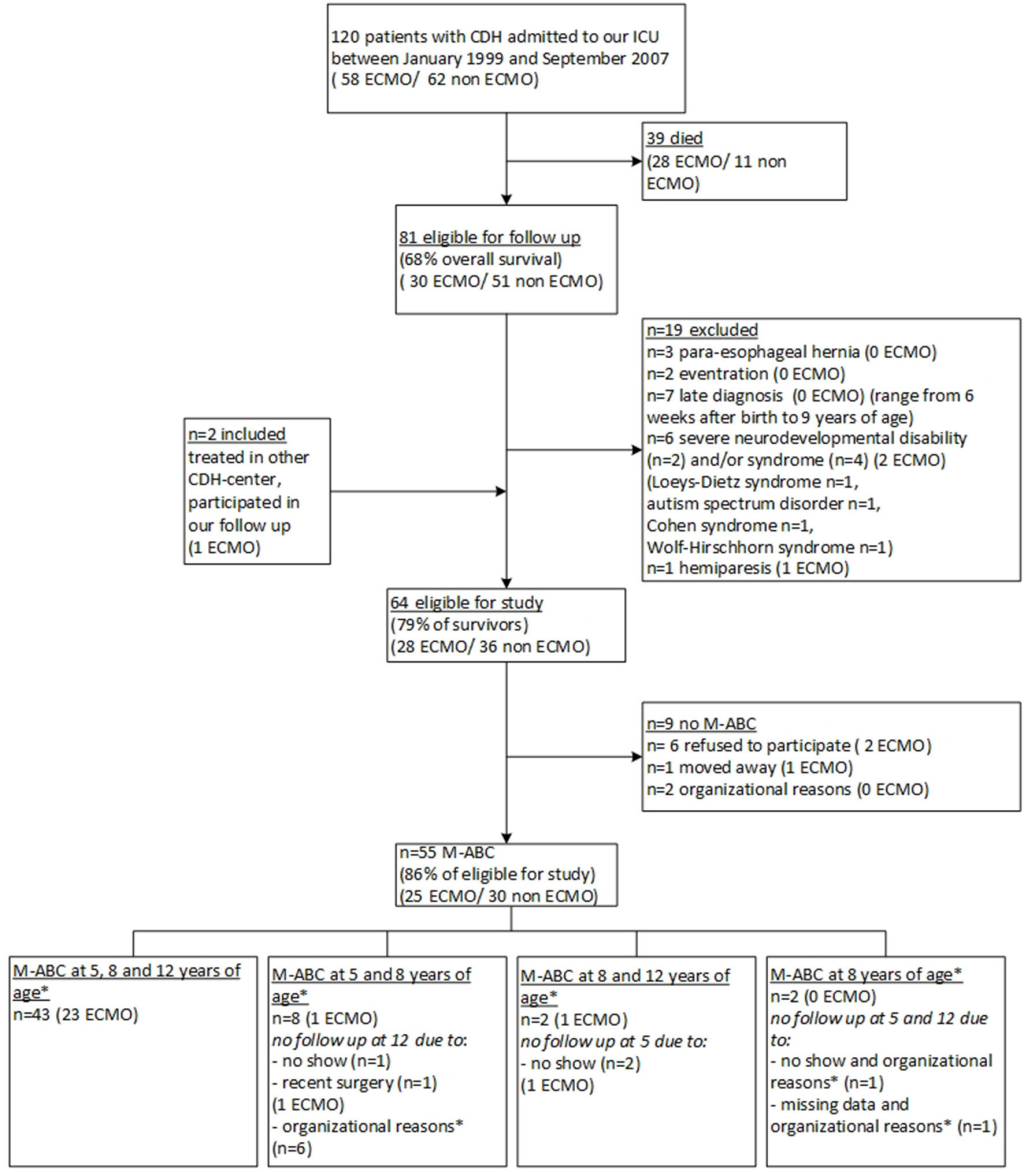
Flowchart of inclusion and exclusion of study participants. CDH, congenital diaphragmatic hernia; ECMO, extracorporeal membrane oxygenation; PMR, psychomotor retardation; M-ABC, Motor-Assessment Battery for Children. * Organizational reasons, in the years 2011–2013, the assessment at 12 years of age was discontinued for non-ECMO-treated patients.

Between January 1999 and December 2007, 120 children born with CDH were admitted to our PICU, of whom 81 (68%) survived to date. After applying the exclusion criteria, 64 patients were eligible for this study, of whom 9 were not assessed for various reasons, resulting in 55 participants. This number includes two participants of our program who were born in another CDH-center (Figure 1). The clinical baseline characteristics of the participants did not differ significantly from those of the non-participants who were lost to follow up, but the frequencies of several characteristics differed significantly between ECMO-treated and non-ECMO-treated participants, such as length of stay and treatment with inhaled nitric oxide (Table 1). At twelve years of age, 52 children (94.5%) had obtained a swimming certificate.

**Table 1:** Background characteristics.

	***Non-ECMO (n = 30) 55%***		***ECMO (n = 25) 45%***		***p–value***	***Non-ECMO n = 30 (55)***	
Boys	17	(57)	18	(72)	0.24	6	(67)
Birthweight, grams	3,000	(1,805–4,900)	3,200	(2,000–3,810)	0.12	3,000	(2,400–3,600)
Gestational age, weeks	39	(35.6–41.4)	39.3	(35.6–41.4)	0.24	39.5	(38.3–39.5)
Inborn	20	(67)	9	(36)	**0.02**	6	(67)
Prenatal diagnosis	20	(69)	10	(40)	**0.03**	6	(67)
Left sided defect	24	(80)	24	(96)	0.08	8	(89)
Age at surgery, days	4	(1–14)	11	(1–42)	**<0.001**	5	(1–20)
Primarily closed	12	(40)	4	(16)	0.05	3	(33)
Initial ventilation, days	10.5	(3–53)	28	(8–146)	**<0.001**	14	(2–64)
Initial PICU stay, days	19	(9–100)	44	(15–153)	**<0.001**	31	(8–99)
Initial hospital stay, days	32.5	(9–113)	77	(22–187)	**<0.001**	31	(15–127)
Cardiac malformations	1	(3)	3	(12)	0.22	0	(0)
Inhaled nitric oxide treatment	16	(55)	24	(96)	**<0.001**	7	(78)
Chronic lung disease	7	(23)	15	(60)	**0.01**	3^a^	(38)
Sepsis during initial hospital stay	3	(10)	9	(36)	**0.02**	3^a^	(38)
Need for ECMO	-		25	(100)		3	(33)
*VA*	-		25	(100)		3	(100)
*Age at start ECMO, hours*	-		14	(2–251)		40	(5–75)
*Time on ECMO, hours*	-		161	(63–369)		236	(83–237)
ISCED level mother							
*Low (ISCED 0–2)*	3	(10)	2	(8)	0.86	0	
*Middle (ISCED 3–4)*	11	(36.7)	8	(32)	0.85	0	
*High (ISCED 5–8)*	12	(40)	11	(44)	0.77	1	(11.1)
*Not available*	4	(13.3)	4	(16)		8	(88.9)

*Data presented as median (range), or n (%). Non-participants: patients lost to follow up or unable to perform M-ABC. ECMO, extracorporeal membrane oxygenation; VA, venoarterial; PICU, pediatric intensive care unit; ISCED, International Standard Classification of Education (19). p-value represents comparison between non-ECMO and ECMO-treated CDH; participants and non-participants were not significantly different and p-values were not shown*.

a *1 missing data. Bold values indicate significant difference between non-ECMO and ECMO-treated CDH patients*.

### Longitudinal Evaluation

**Figure 2:**
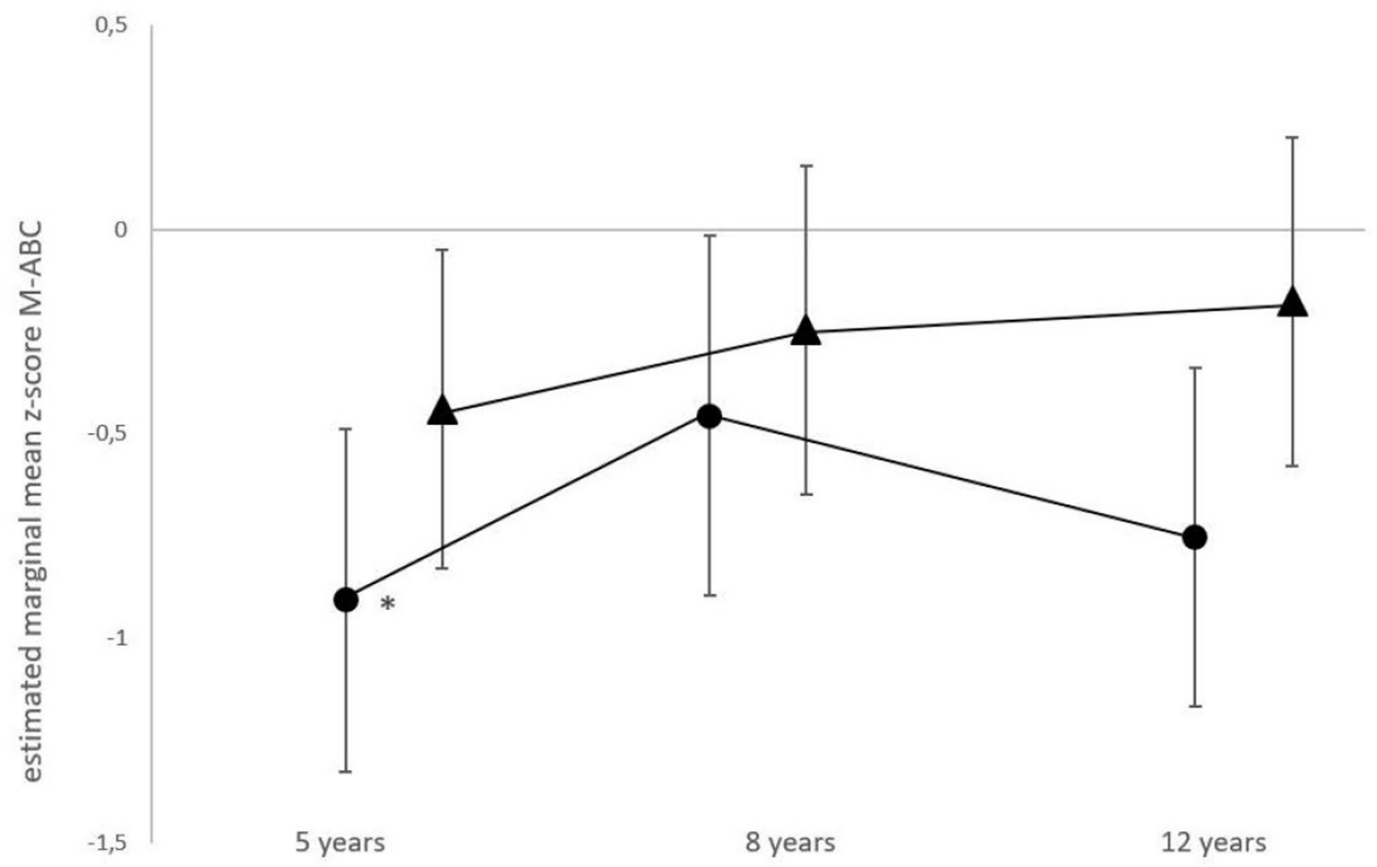
Longitudinal motor function performance in ECMO-treated and non-ECMO-treated children with CDH. Data shown are estimated mean z-scores of the M-ABC with 95% CIs. Circles, ECMO-treated children; triangles, non-ECMO-treated children. *For the ECMO-treated patients, scores differed significantly from age 5 to age 8 years, (*p* = 0.03).

The longitudinal M-ABC results of the total group had improved significantly from age 5 (estimated marginal mean z-score −0.67, 95% CI −0.96 to −0.39) towards age 8 (−0.35, 95% CI −0.65 to −0.05), resulting in a mean difference of 0.32 (*p* = 0.02), followed by a score of −0.46 at age 12 years (95% CI −0.76 to −0.17). For the non-ECMO treated patients, no significant differences in M-ABC scores were found between ages. The estimated marginal mean z-score was −0.44 at 5 years of age (95% CI −0.83 to −0.05), −0.25 at 8 years of age (95% CI −0.65 to 0.16) and −0.18 at 12 years of age (95% CI −0.59 to 0.24). For the ECMO-treated patients, the estimated marginal mean z-score increased significantly from age 5 to age 8 years, from −0.91 (95% CI −1.33 to −0.49) to −0.46 (95% CI −0.90 to −0.02), resulting in a mean difference of −0.45; (*p* = 0.03) and declined at age 12 years (−0.75, 95% CI −1.17 to −0.34) (Figure 2).

### Proportions of Motor Domain Scores Compared to Norm Population

At all three ages, the proportion of ECMO-treated children with a normal total impairment score was significantly lower than in the norm population (chi-square, all *p* < 0.01; Table 2).

**Table 2:** Characteristics and results at follow up.

	**5 years *n* = 51**		**8 years *n* = 55**		**12 years *n* = 45**	
	**Non-ECMO** ***n*** **= 27 (53)**	**ECMO** ***n*** **= 24 (47)**	**Non-ECMO** ***n*** **= 30 (55)**	**ECMO** ***n*** **= 25 (45)**	**Non-ECMO** ***n*** **= 21 (47)**	**ECMO** ***n*** **= 24 (53)**
Boys, (n)	15 (56)	17 (71)	17 (56)	18 (72)	12 (57)	17 (71)
Weight-for-height, z-score	−0.73 (−2.96 to 1.55)	−1.60 (−3.69 to 0.01)	−0.50 (−2.53 to 1.20)	−1.44 (−3.86 to 1.23)	−0.30 (−2.43 to 1.8)	−0.70 (−3.37 to 2.17)
Sports participation, (n)	16 (59)	14 (58)	26 (87)	17 (68)	16 (76)	14 (58)
M-ABC total impairment score						
normal – borderline, > p5 (n)	24 (89)	18 (75)^*^	27 (90)	19 (76)^*^	20 (95)	18 (75)^*^
definite motor problem, p≤5 (n)	3 (11)	6 (25)	3 (10)	6 (24)	1 (5)	6 (25)
M-ABC manual dexterity						
normal – borderline, > p5 (n)	26 (96)	21 (87.5)	28 (93)	21 (84)^∧^	19 (90.5)	18 (75)^*^
definite motor problem, p≤5 (n)	1 (4)	3 (12.5)	2 (7)	4 (16)	2 (9.5)	6 (25)
M-ABC ball skills						
normal-borderline, > p5 (n)	25 (93)	21 (87.5)	28 (93)	22 (88)	17 (81)^*^	22 (92)
definite motor problem, *p* ≤ 5 (n)	2 (7)	3 (12.5)	2 (7)	3 (12)	4 (19)	2 (8)
M-ABC balance skills						
normal – borderline, > p5 (n)	24 (89)	18 (75)^*^	28 (93)	21 (84)^∧^	19 (90.5)	22 (92)
definite motor problem, *p* ≤ 5 (n)	3 (11)	6 (25)	2 (7)	4 (16)	2 (9.5)	2 (8)

*P, percentile. Data shown n (%) or median (range). ECMO, extracorporeal membrane oxygenation. M-ABC, Movement assessment battery for children*.

* *p < 0.01 Chi-square test in comparison with norm values*.

∧ *p < 0.05 Chi-square test in comparison with norm values*.

Concerning subskills, ECMO-treated children performed significantly worse than the norm population on manual dexterity at ages 8 and 12 years (*p* = 0.01 and *p* < 0.001, respectively) and on balance skills at ages 5 and 8 years (*p* < 0.001 and *p* = 0.01, respectively). Ball skills were significantly affected in the non-ECMO treated group at age 12 years (*p* = 0.003) (Table 2).

### Associations

Both in univariate and multivariate analysis, the length of hospital stay was negatively associated with the total z-score of M-ABC (*p* = 0.001 univariate analysis; *p* = 0.004 multivariate analysis), whereas the other variables were not significantly associated with outcome (Table 3).

**Table 3:** Possible determinants of motor performance in the total group of children with CDH.

**Independent variables**	**z-scores of the M-ABC**					
	**Univariate analysis**			**Multivariate analysis**		
	**β**	**95% CI**	***p*** **-value**	**β**	**95% CI**	***p*** **-value**
Birthweight (kg)	0.36	−0.09 to 0.81	0.11	0.31	−0.13 to 0.74	0.16
CLD (yes/no)	−0.45	−0.98 to 0.09	0.10	0.17	−0.51 to 0.84	0.62
Initial hospital stay (days)^a^	**−0.01**	−0.02 to −0.004	**0.001**	**−0.01**	−0.02 to −0.005	**0.004**
Primary closure of defect (yes/no)	−0.09	−0.66 to 0.48	0.76	−0.23	−0.77 to 0.31	0.40
weight–for–height z-score at follow-up	−0.05	−0.21 to 0.12	0.58	−0.13	−0.30 to 0.03	0.10
Sports participation (yes/no)	0.13	−0.17 to 0.42	0.40	0.12	−0.17 to 0.41	0.41

*CLD, chronic lung disease as defined by Jobe and Bancalari (18). Results are based on a general linear model, which has been adjusted for ECMO-treatment and age, as well as for interaction between those two variables*.

a *Significant association. Bold values indicate significant association*.

## Discussion

To the best of our knowledge, this is the first study to longitudinally evaluate the course of motor function in school-aged CDH survivors. We distinguished between those who had been treated with ECMO and those who had not, since it had been shown before that ECMO-treated CDH patients were at risk for impaired motor function (8). Strikingly, at ages 8 and 12 years, the estimated mean z-scores for motor function in non-ECMO-treated participants were not significantly lower than the norm scores, whereas the scores of those treated with ECMO were. At five years of age, scores in both groups were significantly lower than the norm. In multivariate analysis, length of hospital stay was independently associated with poorer motor outcome.

Several groups have studied motor function in children with CDH. Tureczek and co-workers cross-sectionally studied outcome in 3-to-16-year-old non-ECMO-treated children born with CDH. They found that younger children, up till age five years, performed better than older children on adaptive fine and gross motor components, although this finding might have been due to the application of different tests at the various ages (21). Danzer and co-workers longitudinally evaluated motor performance within the first three years of life of children born with CDH, and reported average motor function in the majority of children (10). Church and co-workers performed a retrospective observational study in CDH survivors aged from 4 months to 7.5 years old and found overall motor function to be below average, mainly due to gross motor problems (7). Overall, despite the variability in type of assessment and age of testing, all studies in CDH survivors are consistent in the occurrence of gross motor problems.

Earlier studies in CDH as well as ECMO-survivors revealed need for methadone, CLD and lower observed-to-expected total fetal lung volume as determinants for motor function problems (6–8). In our study, length of hospital was negatively associated with motor function, whereas CLD was not. This association has previously been found in survivors of CDH at toddler age (9, 10). The results of our multivariable analysis suggest that the z-score of M-ABC decreases with 0.01 for every week of initial hospitalization, but our model does not allow to determine a critical threshold of length of hospital stay to predict need of follow-up of motor function. Nevertheless, we propose that risk-stratification should not rely solely on ECMO-treatment, but include length of hospital stay as well. Longer length of stay itself might not be contributive to impaired motor function; it rather reflects severity of disease with underlying problems, such as pulmonary hypertension and failure to thrive.

The five-year-olds in our study appeared to be at risk for poor motor functioning, but motor performance improved thereafter – especially in CDH-survivors who had not received ECMO-treatment. Our data do not allow to conclude what actually has contributed to the improvement, although enrolment in our longitudinal follow-up program, with more timely referral to pediatric physical therapists and active stimulation to sports participation, is likely to have contributed (6, 8, 12, 22).

A few issues relating to this study need to be addressed. First, all but one of the included children underwent laparotomy, which in the study period (1999–2007) was standard of care for surgical correction of CDH. Nowadays, more infants undergo minimal access surgery (23). The question whether gross motor function might be affected by impaired truncal muscle strength after laparotomy has not yet been answered, but deserves further investigation. Second, children with CDH born today are treated with a standardized perinatal protocol introduced in 2008 (24). This protocol resulted in a decline in both mortality and need for ECMO (25, 26). Moreover, CDH is more and more predicted prenatally through standardized ultrasound examination at 20 weeks gestational age. The large majority of the participants in our study were born before the introduction of the protocol and the standardized ultrasound, so that the studied children overall are not fully representative of patients born today.

Although we have previously reported that CDH survivors at school age are at risk for pulmonary morbidity (27), we did not include data concerning lung function. In a randomized controlled trial involving CDH patients with airflow obstruction, we concluded that both exercise tolerance and motor function improved irrespective of intervention, and that parental awareness of reduced exercise capacity rather than specific interventions may have contributed to the improvement (28). This might indicate that lifestyle factors, rather than decreased lung function, contribute to impaired motor function.

Regarding awareness, both the child and its parents tend overestimate the child's motor competence as compared to the results of the M-ABC (29, 30). After having seen their child in a state of critical illness, the urge for well-developed motor function might seem futile to parents (29). Additionally, some parents might consider their child too vulnerable to actively participate in sports. Yet, this underlines the importance of comprehensive counseling of parents.

Moreover, treatment-related causes of motor function impairment might play a role, too. Abnormal cerebral ultrasound findings are not uncommon in this group, especially in ECMO-treated patients, although those findings are not linked to motor development yet (8, 31). Future studies concerning outcome should include the results of close neuromonitoring, as is currently being done in the NEMO-trial (NTR7160), which aims to gain more insight in the physiology of the brain of CDH-patients perioperatively. Also, neuroimaging during the period of critical illness as determinant for future motor development could be of use, to allow detailed risk stratification and prognostication.

Several limitations of our study must be taken into account. First, over the study period, two consecutive versions of the M-ABC were applied. Nevertheless, converting the scores to z-scores allowed comparison between test results (16). Second, this is a single-center study, which limits the transferability of the results. Third, we did not include a score for critical illness, which limited the identification of predictive determinants for impaired motor function. In a previous study by our group, the vaso-active inotropic score (VIS) was found to be predictive of neurocognitive outcome in ECMO-treated patients, and we therefore recommend to take this score into account in further studies concerning development (32). Until 2005 cumulative drug doses were lacking as digital records were unavailable. We were therefore unable to properly retrieve data and calculate VIS for our cohort. Other factors that might have been of interest but were not taken into account in our analyses were social and environmental characteristics, such as time outdoors, availability of toys and space to play, which have been reported to influence motor development (33, 34). We were unable to use those since these data were not collected in the past. However, taking into account the sociodemographic background of the participants (ISCED level mother, Table 1) and the policy of the Dutch government to stimulate sports and swimming classes for low income families, we think that this may not have had an important role. Moreover, we did not investigate cognitive competence and school achievement in this study. Yet, our group has previously studied neurocognitive outcome in school-aged CDH-survivors with and without ECMO, and found problems on several neurocognitive domains. Therefore, persisting problems on several neurodevelopmental domains, such as motor and cognitive function, warrant further evaluation regarding the growing into deficit theory (32).

The strengths of our study can be found in the relatively large cohort with a follow up of 12 years, and the fact that 86% of the eligible candidates actually participated in our follow-up. We found no evidence for selection bias, since the baseline characteristics between the non-participants and participants did not significantly differ.

## Conclusion

In conclusion, children born with CDH are at risk for motor function impairment at the age of five, and impairment may persist up till 12 years of age in those who were treated with ECMO. Length of hospital stay appeared to be an independent risk factor for impaired motor function. Early recognition of motor problems and timely referral to pediatric physical therapists could help prevent worsening. A clinical implication of our findings is that motor function should be monitored up to five years of age in all CDH-survivors. The decision to extend follow up until adolescence should take both need for ECMO-treatment and prolonged hospital stay into account.

## Data Availability Statement

The datasets presented in this article are currently not available. Requests to access the datasets should be directed to h.ijsselstijn@erasmusmc.nl.

## Ethics Statement

The studies involving human participants were reviewed and approved by Medical Ethical Review Committee Erasmus MC Rotterdam. Written informed consent from the participants' legal guardian/next of kin was not required to participate in this study in accordance with the national legislation and the institutional requirements.

## Author Contributions

SdM contributed to the conception and design, acquisition of data, analysis and interpretation of data, and writing of the first draft. MvdC-vZ and HI contributed to the conception and design, acquisition of data, analysis and interpretation of data, writing of the first draft, and critically revising the manuscript. TZ-vdA contributed to acquisition of data and critically revising the manuscript. SC-dO and NvH contributed to interpretation of data and critically revising the manuscript. RW and SG contributed to the conception and design, and critically revising the manuscript. JvR contributed to statistical analysis, interpretation of the data and critically revising the manuscript. All authors approved the final manuscript as submitted and agree to be accountable for all aspects of the work.

## Conflict of Interest

The authors declare that the research was conducted in the absence of any commercial or financial relationships that could be construed as a potential conflict of interest.

## Publisher's Note

All claims expressed in this article are solely those of the authors and do not necessarily represent those of their affiliated organizations, or those of the publisher, the editors and the reviewers. Any product that may be evaluated in this article, or claim that may be made by its manufacturer, is not guaranteed or endorsed by the publisher.

## Abbreviations

CDH: congenital diaphragmatic hernia
CI: confidence interval
CLD: chronic lung disease
ECMO: extracorporeal membrane oxygenation
GLM: general linear model
M-ABC: Movement Assessment Battery for Children
PICU: pediatric intensive care unit
VA: venoarterial.
